# Quantum Osteoimmunology: A Paradigm Shift in Understanding and Influencing Bone–Immune Crosstalk

**DOI:** 10.1002/cmdc.202500307

**Published:** 2025-08-27

**Authors:** Rachel Wei Li, Sara Alzaanin, Zongyou Yin, Paul N. Smith

**Affiliations:** ^1^ School of Medicine and Psycology Australian National University, College of Science, Australian National University Canberra Australian Capital Territory Canberra ACT 2601 Australia

**Keywords:** bioimaging, diagnostics, osteoimmune signaling, quantum dots, quantum mechanics, quantum osteoimmunology, therapeutic delivery

## Abstract

Despite significant advancesin osteoimmunology, the mechanistic underpinnings of immune–skeletal crosstalk remain insufficiently characterized, particularly at the molecular and submolecular scales. The present article introduces quantum osteoimmunology as a novel field of research exploring how quantum mechanical phenomena, such as coherence, tunneling, entanglement, and wavefunction superposition, may influence osteoimmune signaling dynamics. It argues that the current deterministic, temporally linear models of immune activation may overlook the probabilistic and non‐linear nature of molecular events governed by quantum principles. Integrating quantum principles into osteoimmune research could offer new explanatory models for unresolved questions in bone‐immune physiology and pathology. In parallel, the unique photophysical characteristics of quantum nanomaterials, such as size‐tunable emission spectra, high quantum yields, and photostability, present unprecedented opportunities for high‐resolution biomarker detection, enabling real‐time, ultrasensitive diagnostics for osteoimmune pathologies. Moreover, these materials exhibit significant potential for the development of traceable, precision‐targeted therapeutic delivery systems, as well as for high‐resolution in vitro and in vivo bioimaging applications. Ultimately, quantum mechanics holds the potential to revolutionize osteoimmunology—conceptually, by reshaping one's understanding of immune–skeletal interactions at the subatomic level; and practically, by driving innovations in diagnostics, targeted therapeutics, and real‐time molecular imaging.

## Graphical abstract

This article introduces quantum osteoimmunology as a novel field exploring how quantum phenomena may shape immune–skeletal signaling. This approach could help resolve longstanding questions in osteoimmune physiology and pathology. It also highlights the potential of quantum nanomaterials for ultrasensitive diagnostics and precision‐targeted therapeutics in osteoimmune disorders, as well as for high‐resolution bioimaging in both in vitro and in vivo contexts.

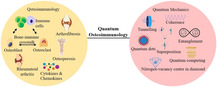

## 1 Introduction

**Figure 1:**
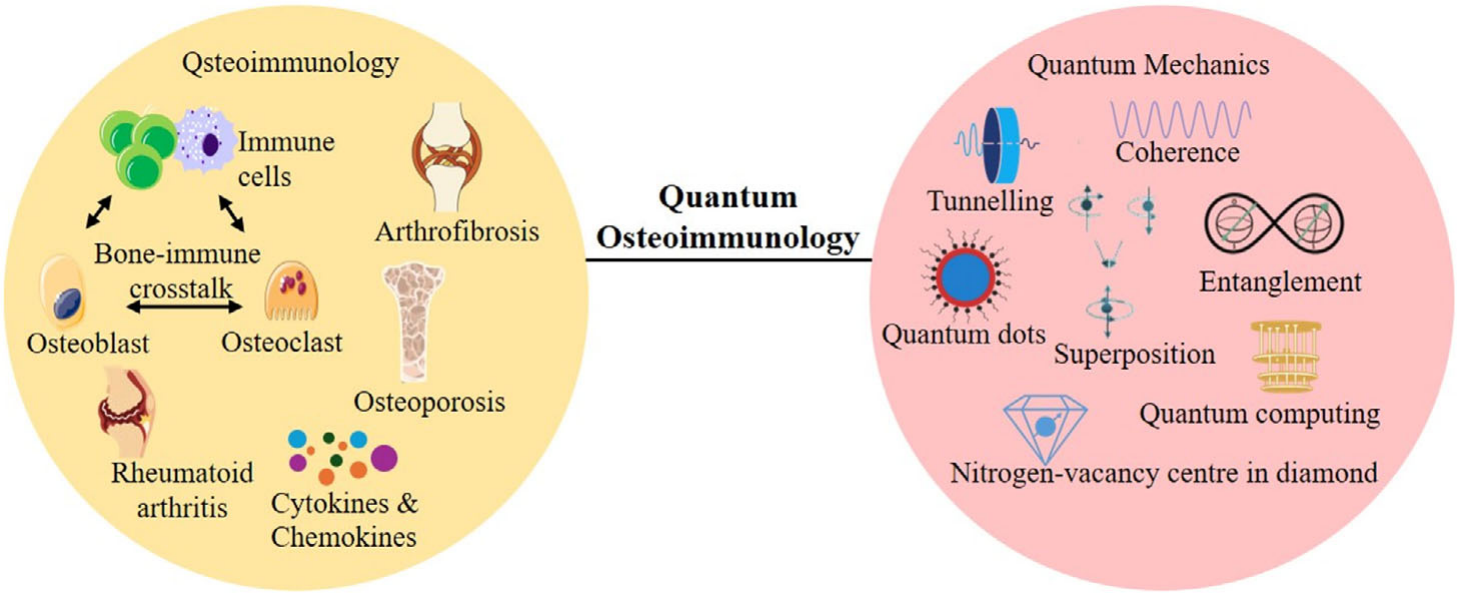
Quantum osteoimmunology: Bridging osteoimmunology and principles of quantum mechanics.

To date, our understanding of the mechanisms governing immune regulation within the skeletal system remains limited, particularly at the molecular and submolecular scales, where quantum‐level interactions may play a role.^[^ ^1^ ^,^ ^2^ ^]^ As a result, we lack a fundamental, mechanistic understanding of the bi‐directional communication between the immune and skeletal systems under both physiological and pathological conditions, particularly in the context of complex disorders such as inflammation‐induced bone loss and autoimmune osteopathies.^[^ ^3^, ^4^, ^5^, ^6^, ^7^, ^8^ ^–^ ^9^ ^]^ This gap in knowledge has hindered progress in accurately identifying the causes of these osteoimmune disorders, as well as in developing effective diagnostic tools and targeted treatments. Hence, we propose a new field of study called “quantum osteoimmunology”, an interdisciplinary domain that bridges the gap between quantum mechanics and osteoimmunology, examining how quantum phenomena, typically observed at atomic and subatomic levels, may influence or be harnessed to better understand and modulate osteoimmunological interactions (**Figure** **1**). The present article explores potential manifestations of quantum effects within osteoimmunology and examines their broader implications for advancing the understanding, diagnosis, and management of osteoimmune disorders.

## 2 Bridging Quantum Mechanics and Osteoimmunology

Osteoimmunologists tend to view the activation of an immune response within bone environments as a temporally linear, deterministic phenomenon.^[^ ^10^ ^]^ In other words, they conceptualize the immune response as if only a single thing is occurring in a predictable, discrete timeframe with a discrete, unchanging momentum in a discrete, single direction, a perspective further reinforced by data visualizations such as heatmaps from transcriptomic and microarray analyses. Such simplification risks ignoring foundational quantum principles, most notably Heisenberg's uncertainty principle, which asserts that there is a fundamental limit to how precisely position and momentum can be known simultaneously,^[^ ^11^ ^]^ and may therefore obscure the dynamic and non‐linear behavior of molecular events at the submolecular level that govern how immunity arises, is modulated, or becomes dysregulated within bone microenvironments.^[^ ^1^ ^,^ ^2^ ^]^

Another conceptual framework from quantum physics that seems relevant is Schrödinger's wave equation, which posits that a particle, rather than being fixed and localized, is better understood as a ‘cloud of probabilities’.^[^ ^12^ ^]^ If we apply this principle to osteoimmune responses, we may consider the trajectory of immune activation in the skeletal system as a probabilistic evolution across space and time, rather than a mono‐directional sequence of events. At the molecular and atomic levels, this suggests that the outcome or ‘direction’ of an immune–bone interaction could follow the path of least action, governed by quantum principles that minimize the difference between potential and kinetic energy. Moreover, modeling osteoimmune interactions, such as the movement of signaling molecules through the osteoimmune network, as probability amplitudes suggests that, much like light waves, these interaction ‘waves’ can constructively or destructively interfere depending on their overlap. Resultingly, they can synergistically amplify or dampen the overall immune response. This analogy opens the door to a Feynman‐style sum‐over‐histories interpretation, wherein the final immune outcome, such as tolerance, inflammation, or bone regeneration, is determined by the absolute square of the sum of complex contributions from all potential molecular pathways, not just one causal chain.

Moreover, quantum osteoimmunology stands to benefit from emerging technologies such as quantum computing and artificial intelligence (AI) to explore and model complex bone–immune interactions at the molecular and submolecular levels, including the probabilistic systems described above. AI systems, including DeepMind's AlphaFold2 and its successors (e.g., AlphaFold Multimer and AlphaFold3), have demonstrated exceptional accuracy in predicting protein folding and protein–protein interactions,^[^ ^13^, ^14^ ^–^ ^15^ ^]^ offering significant utility for modeling key osteoimmune signaling pathways such as RANKL–RANK–OPG complexes. These tools also enable researchers to assess how small mutations in immune or skeletal cell proteins might alter 3D structure and function,^[^ ^16^ ^]^ providing insight into disease mechanisms such as autoimmune osteopathies or inflammation‐induced bone loss. While AI currently leads in predictive structural biology, quantum computing is beginning to show promise in biological applications such as RNA folding and quantum chemistry simulations,^[^ ^10^ ^,^ ^17^ ^,^ ^18^ ^]^ areas that may eventually support osteoimmunological research.

Overall, recognizing the quantum complexity of osteoimmune processes could allow for more nuanced models of osteoimmune signaling, and potentially lead to new ways of predicting, diagnosing, and manipulating immune‐mediated bone diseases at their earliest molecular inception with unprecedented precision. For all these reasons, quantum osteoimmunology is an emerging and exciting field. While the connections between quantum mechanics principles and osteoimmunology remain largely exploratory and theoretical, several key frontiers for future research emerge as particularly promising.

## 3 Pioneering Future Research Frontiers in Quantum Osteoimmunology

### 3.1 Quantum Modeling of Osteoimmune Signaling

**Figure 2:**
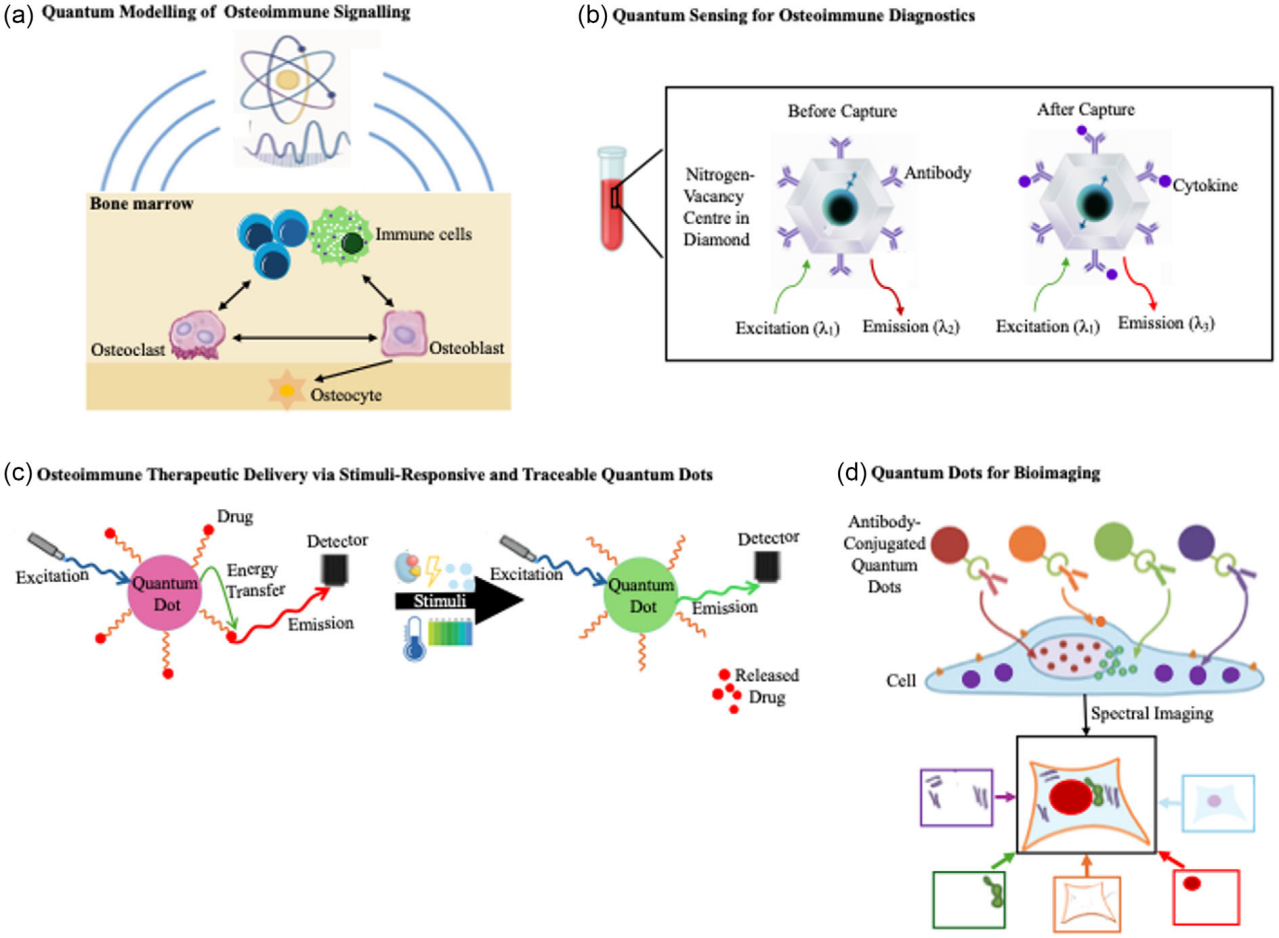
Potential areas of research in the proposed field of quantum osteoimmunology. a) Quantum modeling of osteoimmune signalling pathways to better understand the quantum‐level interactions driving bone–immune crosstalk. b) Quantum sensing of osteoimmune disease‐related biomarkers using the spin‐dependent photodynamic properties of nitrogen‐vacancy (NV) centres in diamond for real‐time, ultra‐sensitive osteoimmune diagnostics. c) Stimuli‐responsive quantum dots engineered to release therapeutic agents at controlled rates or specific anatomical sites in response to localized biochemical cues, such as pH changes, inflammatory cytokines, or enzymatic activity. Simultaneously, their excitation‐dependent fluorescence enables real‐time tracking of both particle localization and drug release. d) Antibody‐conjugated quantum dots facilitate multiplexed cell staining and high‐resolution multicolor imaging.

Bone and immune cells—such as osteoclasts, osteoblasts, macrophages, and T‐cells—communicate through complex molecular mechanisms, including cytokine signaling, RANK/RANKL/OPG interactions, and transcriptional regulation.^[^ ^19^ ^]^ Within this context, quantum osteoimmunology offers the potential to leverage quantum computing to simulate these intricate signaling networks at both molecular and submolecular scales, providing mechanistic insights beyond the reach of classical computational models (**Figure** **2**A). This may include protein folding simulations, dynamic modeling of receptor–ligand binding, and predictive analyses of how genetic or epigenetic variants impact immune–bone crosstalk—particularly in the pathogenesis of disorders such as osteoporosis, rheumatoid arthritis, and arthrofibrosis. Notably, quantum phenomena such as tunneling—the phenomenon by which particles can pass through a potential energy barrier, even if they do not have enough energy to overcome it classically^[^ ^20^ ^]^—and coherence—the phenomenon by which particles maintain a fixed and predictable phase relationship with each other so they act in a correlated, unified way rather than as isolated parts^[^ ^21^ ^]^—may be especially relevant for understanding the energetic and probabilistic dynamics of interactions between osteoimmune receptors (e.g., RANK, IL‐6R) and their corresponding ligands or antigens. These insights could accelerate the identification of molecular targets involved in bone resorption, inflammation, or fibrosis, thereby supporting the design of targeted therapies for osteoimmune conditions. Furthermore, quantum coherence may help explain the mechanisms underlying immunological memory to autoantigens, pathogens, or vaccine antigens within bone‐resident immune cells.^[^ ^10^ ^]^ Nanoscale coherence phenomena could, for example, enhance mitochondrial energy efficiency via coherent electron transport, thereby supporting the long‐term survival of memory cells; improve the fidelity of intracellular signaling through coherent protein conformational transitions; and stabilize epigenetic memory states through proton tunneling in DNA‐ and chromatin‐modifying enzymes. Collectively, these processes may contribute to the preservation of stable transcriptional programs required for long‐term immunological memory in the specialized microenvironments of bone marrow niches.

### 3.2 Quantum Sensing for Osteoimmune Diagnostics

Advancements in quantum sensing technologies—such as the nitrogen‐vacancy centres in diamond and quantum dots, the latter being nanoscale semiconductor particles—offer a novel opportunity for real‐time, ultra‐sensitive detection of molecular and cellular events relevant to bone–immune health, with both systems being renowned for their exceptional optical properties.^[^ ^22^ ^,^ ^23^ ^]^ In the context of quantum osteoimmunology, these sensors—functionalized with specific peptides, ligands, or antibodies—hold significant potential for identifying and monitoring key biomarkers such as pro‐inflammatory cytokines (e.g., IL‐6, TNF‐*α*), bone turnover markers (e.g., CTX, P1 NP), and autoantibodies relevant to bone and immune disorders in real time from biological fluids like blood, synovial fluid, or bone marrow aspirates (Figure 2B). This would enable the early detection and continuous monitoring of pathological changes associated with conditions such as rheumatoid arthritis, osteoporosis, arthrofibrosis, or even skeletal metastases in cancer, as well as the tracking of therapeutic responses over time. Unlike conventional diagnostics, quantum sensors operate with exceptional sensitivity at the nanoscale,^[^ ^24^ ^]^ allowing for the identification of molecular signatures well before they manifest as clinical symptoms or radiographic changes. Additionally, quantum dots have the ability to support multiplexed detection, for example, the labeling of multiple targets with different colors in a single assay, due to their size‐ and composition‐dependent tunable emission spectra, high photostability, and surface versatility.^[^ ^25^, ^26^ ^–^ ^27^ ^]^ This makes them highly efficient for comprehensive diagnostic profiling. Furthermore, some advanced quantum dots are even stimuli‐responsive, enabling them to signal pathological environments like sites of inflammation or bone resorption.^[^ ^28^, ^29^, ^30^ ^–^ ^31^ ^]^ Moreover, the integration of quantum sensing with AI‐powered analytics could support personalized disease monitoring and timely therapeutic interventions, improving clinical outcomes in chronic osteoimmune disorders.

### 3.3 Quantum Dots in Osteoimmune Therapeutic Delivery

Quantum dots have demonstrated promising biomedical applications, including in therapeutic delivery systems.^[^ ^32^, ^33^, ^34^ ^–^ ^35^ ^]^ Within the emerging field of quantum osteoimmunology, quantum dots may serve as powerful tools to enhance the precision, monitoring, and control of therapies targeting bone–immune interactions. Quantum dots can be loaded with drugs through a variety of methods, such as dispersion, adsorption, dissolution, and coupling.^[^ ^36^ ^,^ ^37^ ^]^ Their surfaces can be further functionalized with bone‐ or immune‐targeting ligands, antibodies, or peptides, enabling precise delivery to sites of bone remodeling, inflammation, or cellular cross‐talk between osteoblasts, osteoclasts, and immune cells. Additionally, their intrinsic fluorescent properties allow for real‐time tracking of osteoimmune‐targeted biologics as they distribute through bone tissue and immune‐related compartments.^[^ ^38^ ^,^ ^39^ ^]^ This could provide critical insights into therapeutic localization, uptake, and efficacy—particularly in conditions like osteomyelitis, arthrofibrosis, and autoimmune bone diseases, where immune activity is spatially heterogeneous and dynamically regulated. Additionally, as illustrated in Figure 2C, the inherent quantum properties of these nanoparticles may be harnessed to design stimuli‐responsive delivery platforms capable of releasing therapeutic agents—such as cytokine inhibitors, antigens, or bone‐regenerative molecules—at precise rates or anatomical sites in response to localized biochemical signals (e.g., pH shifts, inflammatory cytokines, or enzymatic activity).^[^ ^28^ ^–^ ^31^ ^]^ Such smart delivery systems could significantly improve treatment specificity and reduce systemic side effects in chronic osteoimmune disorders.

### 3.4 Quantum Dots for Bioimaging

Furthermore, as illustrated in Figure 2D, quantum dots hold significant potential for both in vivo and in vitro bioimaging because, as previously mentioned, they have exceptional optical properties—such as photostability, high brightness, and tunable emission spectra.^[^ ^23^ ^,^ ^36^ ^]^ They can be functionalized with ligands, antibodies, or peptides to label and track osteoblasts, osteoclasts, and immune cells, as well as to monitor molecular signals like cytokines involved in bone‐immune regulation. Therefore, they can enable real‐time tracking of immune cell migration, bone remodeling activity, and localized immune responses, offering valuable insights in conditions like osteoporosis and inflammatory bone diseases.

Figure 2 outlines key future research directions within the proposed interdisciplinary field of quantum osteoimmunology.

## 4 Potential Challenges and Strategies

Despite its conceptual appeal and potential clinical relevance, the development of quantum osteoimmunology as a scientific field faces several formidable challenges. Chief among these is the difficulty of experimental validation and reproducibility, which contributes to quantum biology remaining a controversial and speculative domain, with many proposed phenomena still lacking independent verification. This may impede progress in bridging quantum mechanical principles with osteoimmunology. Another significant concern is biocompatibility and toxicity in relation to quantum dots and other nanomaterials. Many quantum dots contain heavy metals such as cadmium, which pose potential risks for in vivo use. A promising avenue to overcome this challenge is the development of carbon‐based (e.g., graphene quantum dots) and silicon‐based quantum dots, which have demonstrated lower toxicity profiles while retaining desirable optical and electronic properties.^[^ ^24^ ^,^ ^39^ ^]^ Ongoing surface modification strategies, such as PEGylation, ligand exchange, and biomimetic coatings—can further improve safety and targeting efficiency.^[^ ^37^ ^]^

Compounding these technical challenges is the epistemological divide between quantum physics and biomedical sciences. Most current osteoimmunological models are grounded in classical biology and deterministic frameworks that lack the capacity to accommodate quantum probabilism, coherence, and tunneling. This disciplinary gap is compounded by the scarcity of researchers trained in both quantum theory and biomedical research, limiting effective cross‐pollination of ideas. To address this, we propose the establishment of interdisciplinary research consortia and training programs that unite quantum physicists, biologists, immunologists, and materials scientists. Programs modeled after initiatives like the Human Frontier Science Program or the NSF's Quantum Leap Challenge Institutes could catalyze collaborative infrastructure and generate shared conceptual vocabularies.^[^ ^40^ ^]^

Furthermore, the field must navigate cultural resistance within the biomedical sciences. As Kuhn famously noted, scientific revolutions often face initial resistance due to entrenched paradigms.^[^ ^41^ ^]^ To facilitate cultural adoption, the field should prioritize publishing high‐quality, mechanistic studies in interdisciplinary journals and engage in scientific diplomacy, including invited symposia, policy white papers, and stakeholder workshops, to raise awareness and foster receptivity.

In summary, while the field of quantum osteoimmunology presents significant conceptual and practical challenges, it will provide a transformative lens through which to understand, predict, and therapeutically influence bone–immune communication, unlocking possibilities for precision diagnostics, quantum‐designed biologics, and real‐time, molecular‐level disease interception.

## Conflict of Interest

The authors declare no conflict of interest.
